# A Novel Nanocone Cluster Microstructure with Anti-reflection and Superhydrophobic Properties for Photovoltaic Devices

**DOI:** 10.1186/s11671-018-2754-4

**Published:** 2018-10-23

**Authors:** Jing Ma, Yuanfei Ai, Lei Kang, Wen Liu, Zhe Ma, Peishuai Song, Yongqiang Zhao, Fuhua Yang, Xiaodong Wang

**Affiliations:** 1 Engineering Research Center for Semiconductor Integrated Technology, Institute of Semiconductors, Chinese Academy of Science, Beijing, 100083 China; 2 College of Materials Science and Opto-Electronic Technology, University of Chinese Academy of Sciences, Beijing, 101408 China; 3 Institute of Fundamental and Frontier Sciences, University of Electronic Science and Technology of China, Chengdu, 610054 China; 4 University of Chinese Academy of Sciences, Beijing, 101408 China; 5 State Key Laboratory for Superlattices and Microstructures, Institute of Semiconductors, Chinese Academy of Sciences, Beijing, 100083 China; 6 School of Microelectronics, University of Chinese Academy of Sciences, Beijing, 101408 China

**Keywords:** Nanostructure, PDMS, Anti-reflection, Superhydrophobic

## Abstract

As three-dimensional (3D) nanostructures can significantly improve the absorption capacity of photons, it is widely used in various photovoltaic devices. However, the high-cost and complex preparation process of traditional 3D nanostructures restricted its development greatly. In this paper, a new type of nanocone cluster microstructure was prepared on polydimethylsiloxane (PDMS) substrate by using a simple template process. This novel nanocone cluster microstructure can significantly improve the light transmittance and reduce the light reflection, showing superior anti-reflection property. In the whole range of visible band, the nanocone cluster microstructure effectively reduces the reflectivity of the light, so that it remains below 3.5%. In addition, this kind of cluster microstructure showed excellent superhydrophobic property and self-cleaning ability with the contact angle of 151°.

## Introduction

Photovoltaic devices are promising candidates for renewable and sustainable solar energy [1]. But low light absorption coefficient and low efficiency of the device restrict its development greatly. Anti-reflection (AR) film [2, 3], which utilizes light management strategies to reduce reflection within a relatively thin layer of active materials, is considered an effective way for the photovoltaic devices [4, 5]. AR properties can be achieved by introducing micro/nanostructures on flat films [4]. So far, a variety of anti-reflection structures have been reported, such as nanoholes [6–8], nanowires [9], nanoparticles [10], and nanocones [11, 12].

Superhydrophobic property is another key ingredient to determine the efficiency of photovoltaic devices. According to the report, the efficiency of solar cells could decrease by 50% due to dust accumulation every year [4, 13]. Therefore, it is highly desirable to propose a method to keep the surface of photovoltaic devices unstained [4]. Superhydrophobic surface possesses good self-cleaning property, which can be used to remove undesirable contaminants from the surface of photovoltaic devices easily [14], an economic way to solve the problem mentioned above.

However, it is difficult to develop a nanostructured film with both anti-reflection property and superhydrophobic property at the same time. Since typical superhydrophobic property is usually achieved on a rough surface. Meanwhile, rough structured surfaces often suffer from strong scattering or diffraction effects, thus inducing large loss of light [4, 15]. Therefore, the researches about the multifunctional films with superhydrophobic and anti-reflection properties are rarely reported. In 2012, Kyu Back Lee et al. [14] fabricated nanostructures with a RIE method on quartz surfaces with self-cleanability and anti-reflectivity. Here, they used quartz as the substrate, which was not flexible and the cost of RIE process was also very high. In 2017, Fan et al. [16] presented a nanocone array anti-reflection film with superior superhydrophobicity, but the reflectance in the long wavelength was unsatisfied. Therefore, it is imperative to develop environment-friendly and simple flexible nanostructure films with anti-reflection and superhydrophobic properties [4].

In this paper, we demonstrated a new type of nanocone cluster microstructure prepared on PDMS substrate by using a simple template process. This novel nanocone cluster microstructure can significantly improve the light transmittance and reduce the light reflectivity, which can be used in photovoltaic devices to improve the efficiency. Meanwhile, it possesses superior superhydrophobic property, with a water contact angle (CA) of 151°. This unique property leads to a self-cleaning function and water-repellent feature [16]. In addition, PDMS is an environment-friendly, flexible, and highly transparent material, which is also good for the improvement of light transmittance [4, 17].

## Methods

### Preparation of Nanocone Cluster Microstructures

**Fig. 1:**
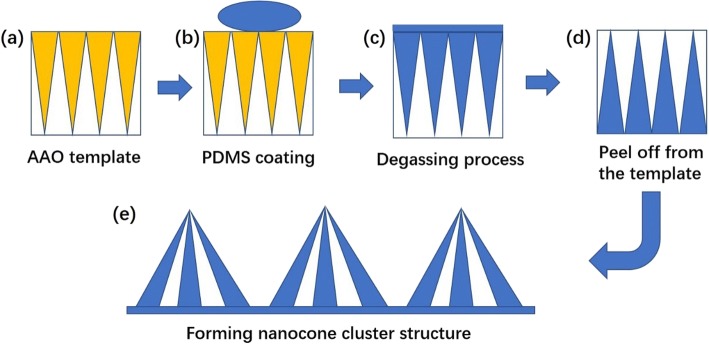
**a**–**e** The schematic fabrication process of nanocone cluster microstructures

**Fig. 2:**
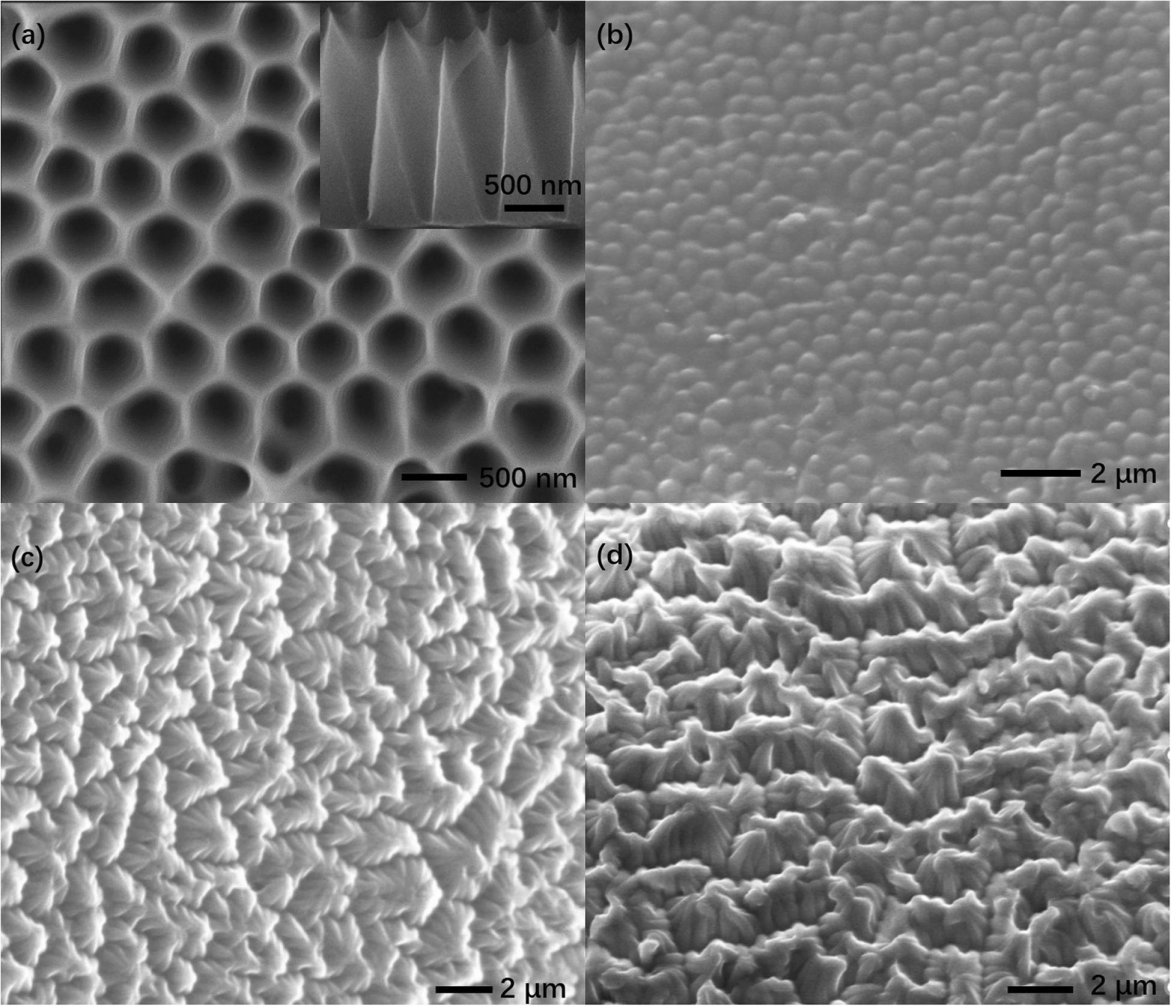
SEM images of **a** V-shape AAO template and **b**–**d** PDMS nanocones with aspect ratios of 1, 2, and 3

Anodized aluminum oxide (AAO) template can be obtained by multistep anodization using an acidic solution and proper DC voltage, followed by a wet etching process [11, 16, 18, 19]. Here, we used three templates with different aspect ratios (AR, defined by height of nanocones over periodicity) of 1, 2, and 3 to investigate the effect of nanocone size on its performance. The pitch of templates was 450 nm, and the height was 450 nm, 900 nm, and 1350 nm corresponding to the aspect ratio of 1, 2, and 3. The small pitch of the template was benefit for the preparation of the cluster structure because smaller pitch leads to larger aspect ratio. The structure with larger aspect ratio usually owns huge system energy. In order to maintain the stability of the structure, some of the system energy will be released during the curing process [20]. Thus, the single nanocone was more easily to incline and aggregate together to form nanocone cluster microstructures after drying. AAO template was cleaned by acetone, ethanol, and distilled water, followed by a spin coating of anti-sticking agent (GL-AAC, GermanLitho). Then, the PDMS solution (GL-ML CURE, GL-ML BASE, GermanLitho, 10:1 ratio) was drop-cast on the V-shape template and the sample was pumped in a vacuum vessel to remove air bubbles in the PDMS solvent, followed by a curing process at 75 °C for 4 h as shown in Fig. 1b, c [16]. Finally, PDMS nanocone films with a thickness of 0.3 mm were peeled off directly from the V-shape AAO template when the sample cooled down to room temperature. As the pitch between each cone is very small and the height is very high, nanocones will be inclined to the side at the moment when the PDMS film is peeled off from the template, resulting in 6–8 cones aggregating together and forming nanocone cluster microstructures after drying (Fig. 2c).

### Characterizations

The morphology analysis of as-prepared products was characterized by scanning electron microscopy (SEM, FEI NanoSEM650, Hillsboro, OR, USA) [21]. The hydrophobic performance of the products was measured by a JC2000D water contact angle tester (Zhongchen Digital Technic Apparatus Co., Ltd., Shanghai, China). The optical property was measured by a Varian Cary5E spectrophotometer in the range of 400–1100 nm.

## Results and Discussion

Figure 1 demonstrates the fabrication procedures of the nanocone cluster microstructure. V-shape AAO was used as the template. The anti-sticking agent (GL-AAC, GermanLitho) was spin coated on the AAO template to make following procedures more easily. Then, the PDMS solution (GL-ML CURE, GL-ML BASE, GermanLitho, 10:1 ratio) was drop-cast on the V-shape template followed by a degassing process and then cured at 75 °C for 4 h, as shown in Fig. 1b, c. The PDMS film was peeled off from the V-shape AAO template after the sample cooling down to the room temperature. The structure was thought to be vertical, just as shown in Fig. 1d. However, as the pitch between each cone is very small and the height is very high, nanocones will be inclined to the side and aggregated together in order to reduce the surface energy, thus forming the nanocone cluster microstructure (Fig. 1e). The aggregation of nanocones could be described in terms of two processes: fractal percolation and general Brownian movement. In the beginning, all the particles involved in PDMS solutions moved chaotically over the lattice points in fractal Brownian motion. When two particles met, they formed stable doublets, lost their mobility, and became the nuclei for the aggregates. When wandering particles approached cells next to aggregates, they were captured and became elements of the aggregate. Thus, more and more free particles were bound into an aggregate and form nanocone cluster microstructure [22].

Figure 2 represents the SEM images of the V-shape AAO template and PDMS nanocones with aspect ratios of 1, 2, and 3 after template process. Figure 2a and the inset show the top view and cross view of the template with the pitch and height of 450 and 900 nm, respectively. Figure 2b–d displays the SEM image of nanocone microstructures with aspect ratios of 1, 2, and 3. From the images, we can learn that the morphology was still separate nanocone microstructures after template process with template of aspect ratio 1. Figure 2c, d shows the image of the nanocone cluster microstructures with aspect ratios of 2 and 3 templates. The nanocone cluster microstructure is composed of several nanocones, forming a cluster structure with good hydrophobicity and anti-reflectance. It can be seen that about 6–8 single nanocones aggregating together to form nanocone cluster microstructures with the diameter of 950 nm and the height of 650 nm, as shown in Fig. 2c. While the nanocone cluster microstructures formed in Fig. 2d is composed of over 10 separate nanocones. The results obtained in Fig. 2c, d can be explained as follows: the morphology of PDMS structure is related with height and pitch of the structure. In the beginning, the angle between the structure and the substrate (we call it sidewall angle [20]) was vertical. As the height of the structure increases, the sidewall angle of the structure also increased because the nanocones far from the origin of the structure were more easily inclined [20]. And because of the small pitch of the structure, the inclined nanocones begin to aggregate together to form nanocone cluster microstructures.

**Fig. 3:**
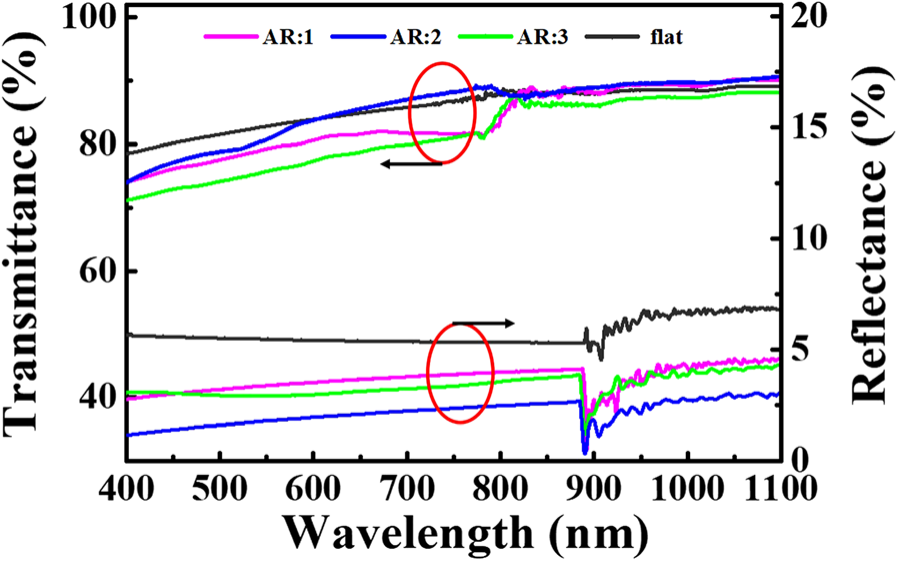
Reflectance and transmittance measurements of the PDMS films with and without nanocone cluster microstructures

In order to investigate the optical properties of the patterned film, optical reflectance and transmittance spectra were measured at normal incidence and flat PDMS film was also tested for reference, as shown in Fig. 3. Apparently, the reflectance of the patterned film was significantly reduced as compared with the flat PDMS film in a broad wavelength range. Samples with nanocone aspect ratio of 2 exhibit excellent anti-reflection performance with the reflectance of below 3.5% in a wavelength range of 400–1100 nm [4], while the reflectance keeps below 5 and 4.5% for nanocone aspect ratio of 1 and 3, respectively. The low reflectance of the patterned film is originated by the gradual change in the refractive index between the air and PDMS surfaces obtained by the nanocone cluster microstructures [23, 24]. And this is also the evidence of showing that aggregated nanocone cluster microstructure has better performance in reducing reflection than separated nanocones.

Figure 3 also displayed the transmittance of PDMS films with and without nanostructures measured as a function of wavelength. From Fig. 3, we can learn that the surface reflectance of the PDMS film with nanocone cluster microstructures keeps higher transmittance values in long wavelength range compared to the flat PDMS films. PDMS films with aspect ratio of 2 show the best transmission of light in the long wavelength. This is because higher aspect ratio nanocones will provide a smoother gradient of effective refractive index, increase light scattering, and suppress front side reflectance. However, too high aspect ratio structure has lower specific surface area, which is not good for light transmittance. That is why we choose PDMS films with aspect ratio 2 for further studies.

**Fig. 4:**
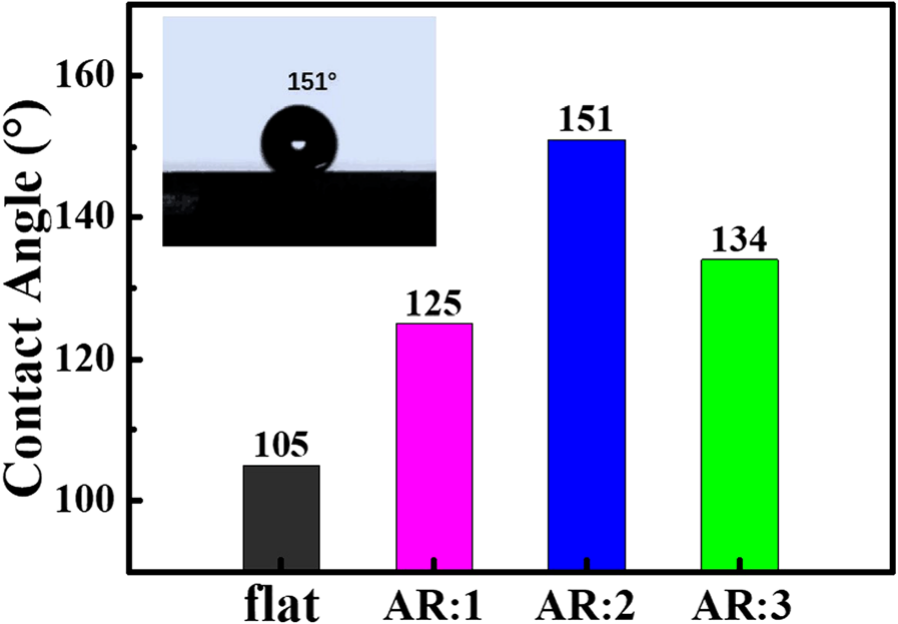
The water contact angles of PDMS films with different aspect ratios

**Fig. 5:**
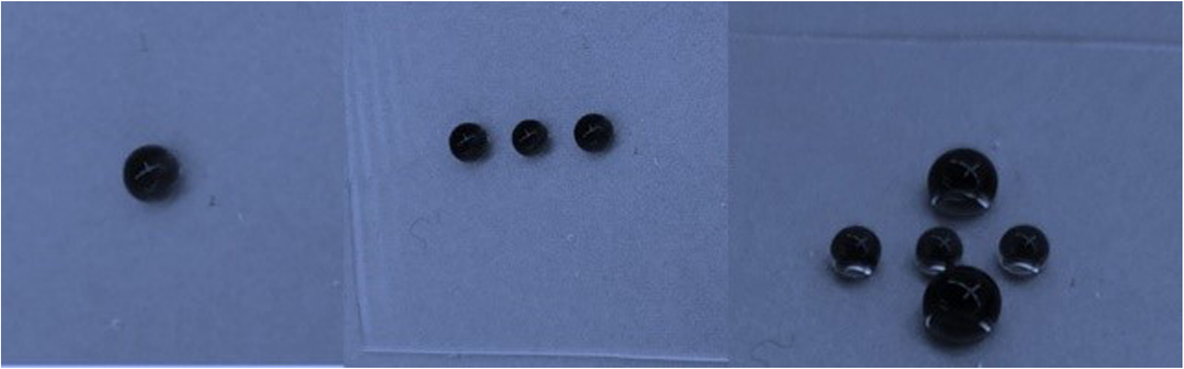
Water droplets on a large surface of the superhydrophobic PDMS film

Figure 4 shows water CAs of PDMS films with different nanocone aspect ratios. The flat film shows hydrophobic property with a water CA of 105° because of the large bond energies of C–H [25]. Films with micro/nanostructures would improve the hydrophobic characteristics with larger CAs compared with the flat one [5]. It is easier to see that the contact angle increases first and then decreases with nanocone aspect ratio increases, and films with aspect ratio 2 nanocones showing a contact angle up to 151°, which satisfies the critical condition of superhydrophobicity (Fig. 4). And from the histogram, we can also learn that aggregated nanocone cluster microstructures have lager CAs than separated nanocone microstructures. Figure 5 displayed water droplets on a large surface of the superhydrophobic PDMS films, also demonstrating superior superhydrophobicity. This phenomenon can be explained by Cassie’s equation [20, 26–28]:

$$ {\mathrm{cos}\uptheta}_{\gamma }={f}_1\cos {\theta}_1-{f}_2 $$

Here, θ_*γ*_ and θ_1_ represent the CA of PDMS films with and without surface structures. So, *θ*_*γ*_= 151°and *θ*_1_= 105°. *f*_1_ is the ratio of surface structure area on a solid-liquid interface, and *f*_2_ is the area fraction of air on the solid-liquid interface.

Moreover,

$$ {f}_1+{f}_2=1. $$

We can calculate that *f*_1_ is 0.169 and *f*_2_ is 0.831.

From the above calculation, we can learn that the water droplets are mainly in contact with air in the solid-liquid interface, which is why the nanocone cluster microstructure we prepared has excellent hydrophobic performance. The improved hydrophobicity also enhanced the self-cleaning effect and water-repellent property significantly, which decreases the cleaning cost of the device greatly and makes it a good candidate in photovoltaic device applications [4, 5, 28].

From the above “Results and Discussion” section, we can learn that the aggregated nanocone cluster microstructure exhibits lower reflectance and larger CAs compared with separated nanocone microstructure. This is also consistent with the conclusion reported in the literature [20]. So far, the nanocone microstructure can be transferred to other substrates like silicon and sapphire. And it has been applied to photovoltaic devices. As the morphology of the nanocone cluster microstructure is hard to control during the transfer process, it is difficult to transfer this kind of cluster microstructure to other substrates at present. But with the development of nanofabrication facilities, the structure can be used in various fields through technologies like nanoimprint lithography and electron beam lithography.

## Conclusions

In summary, we have demonstrated a new type of nanocone cluster microstructure prepared on PDMS substrate by using a simple template process. This novel nanocone cluster microstructure can significantly improve the light transmittance and reduce the light reflection, thus improving the performance of photovoltaic devices. In the whole range of visible band, when the light incident was at the normal angle, the nanocone cluster microstructure effectively reduces the reflectivity of the light, so that it remains below 3.5%. In addition, this kind of cluster nanostructure showed excellent hydrophobic property and self-cleaning ability as the CA is 151°. These results suggest that this kind of nanostructured PDMS thin films developed here is an ideal candidate for future low-cost high-performance energy collection and optoelectronic devices [29].

## Abbreviations

3D: Three-dimensional
AAO: Anodized aluminum oxide
AR: Aspect ratio
CA: Contact angle
PDMS: Polydimethylsiloxane
SEM: Scanning electron microscopy
